# Ocotillol Enhanced the Antitumor Activity of Doxorubicin via p53-Dependent Apoptosis

**DOI:** 10.1155/2013/468537

**Published:** 2013-06-13

**Authors:** Hongbo Wang, Pengfei Yu, Jing Bai, Jianqiao Zhang, Liang Kong, Fangxi Zhang, Guangying Du, Shiqian Pei, Lixia Zhang, Yongtao Jiang, Jingwei Tian, Fenghua Fu

**Affiliations:** ^1^Key Laboratory of Molecular Pharmacology and Drug Evaluation, Ministry of Education of China, School of Pharmacy, Yantai University, Yantai 264005, China; ^2^State Key Laboratory of Long-Acting and Targeting Drug Delivery Technologies, Luye Pharma Group Ltd., Yantai 264003, China; ^3^Center of Basic Medicine, Binzhou Medical College, Yantai 264005, China

## Abstract

The use of doxorubicin (Dox) was severely constrained by dose-dependent side effects, which might be attenuated by combining a “sensitizer” to decrease its cumulative dosage. In this study, it was investigated whether ocotillol could enhance the antiproliferation activity of Dox. MTT assays and xenograft tumor model were firstly conducted to evaluate the effect of ocotillol on the antitumor activity of Dox. Flow cytometry and Hoechst staining assays were then performed to assess cell apoptosis. Western blot and real-time PCR were finally used to detect the expression of p53 and its target genes. Our results showed ocotillol to enhance Dox-induced cell death in p53 wild-type cancer cells. Compared with Dox alone, Dox with ocotillol (Dox-O) could induce much more cell apoptosis and activate p53 to a much greater degree, which in turn markedly increased expression of proapoptosis genes. The enhanced cytotoxic activity was partially blocked by pifithrin-**α**, which might be through attenuating the increased apoptosis. Furthermore, ocotillol significantly increased the antitumor activity of Dox in A549 xenograft tumor in nude mice. These findings indicated that ocotillol could potentiate the cytotoxic effect of Dox through p53-dependent apoptosis and suggested that coadministration of ocotillol with Dox might be a potential therapeutic strategy.

## 1. Introduction

Cancer was one of the leading causes of death in the world [1], and chemotherapy, rather than surgery or radiotherapy, remained the most effective strategy for prolonging survival and improving cancer patients' quality of life [2, 3]. Doxorubicin (Dox) was a potential chemotherapeutic agent, and its use was part of several standard regimens for different cancers [4, 5]. Although Dox had been shown to exert robust antitumor activity, its effectiveness was often restricted by drug-resistance and dose-dependent side effects, especially the Dox-induced cardiomyopathy [5, 6]. Therefore, novel combination chemotherapeutic strategy, in which one novel compound was added to increase the therapeutic index of Dox, would definitely benefit the cancer patients.

The mechanisms of action of Dox in inducing apoptosis in cancer cells via p53 activation had been widely investigated [7]. As the gatekeeper of the genome, the tumor repressor p53 was rapidly activated upon Dox-induced DNA damage and functioned as a transcription factor in regulating downstream target genes, such as *PUMA*,* PIG3*, and *BAX* [8, 9]. The novel compounds, which could potentiate the p53 activation after cotreatment with Dox, might work as one “sensitizer” to reduce the “toxic dosage” to “subtoxic dosage” for Dox and prevent the lethal cardiomyopathy. Indeed, several agents, such as IFN-*α*, inorganic phosphate, and curcumin, were reported to potentiate the antitumor activity of Dox via p53 activation [10–12].

**Figure 1:**
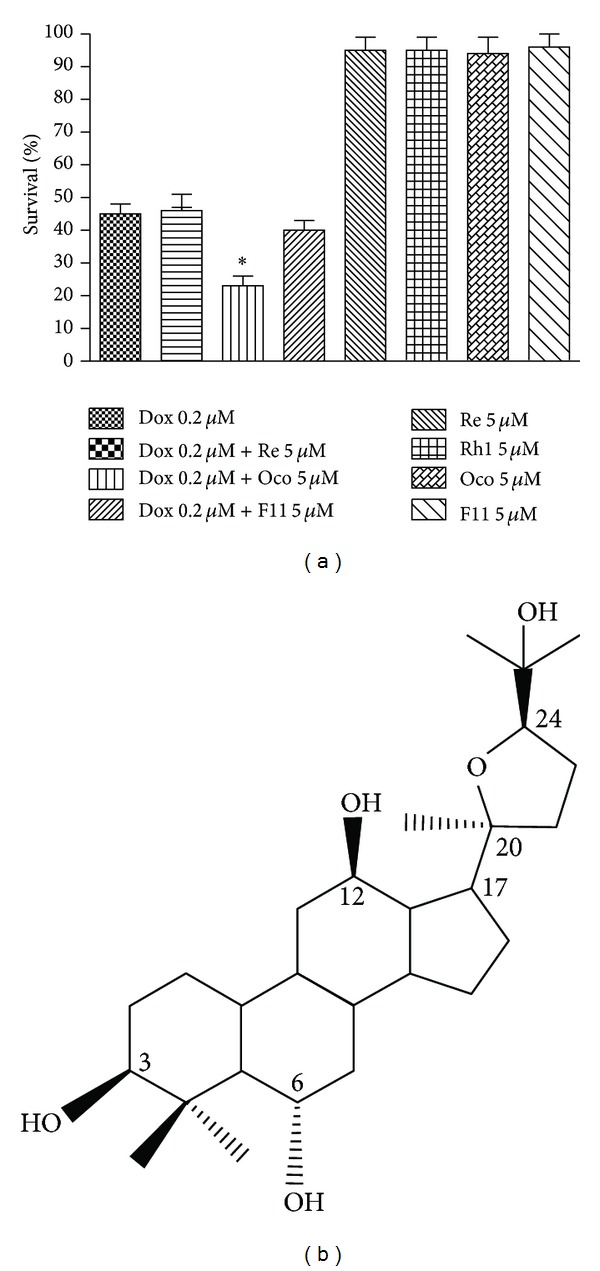
The effects of four ginsenosides on the cytotoxic activity of Dox in A549 cells. (a) A549 cells were seeded into 96-well plate and treated with Dox (0.2 *μ*M), ginsenoside (5 *μ*M), or the combination as indicated for 72 h. The cell viability was detected by MTT assay after 72 h incubation. (b) Chemical structure of ocotillol. *P < 0.05, compared with Dox group.

Natural sources, including plants, microorganisms, and halobiotics, provide rich resources for discovery of novel drugs [13]. Ginseng had a wide range of pharmacological actions, such as neuroprotective, cardioprotective, antioxidant, and anticancer properties, which were primarily attributable to the presence of different ginsenosides [14, 15]. Among which, Ginsenosides Rg3 and Rh2 were reported to enhance antitumor activity and/or decrease the toxic effects of several cytotoxic drugs, such as Dox, paclitaxel and cyclophosphamide [16–19]. As part of our continuing effort to discover novel agents from natural products to improve the therapeutic outcome of Dox, we tested the effects of several ginsenosides on its antitumor activity using MTT assay. Ocotillol (Figure 1(b)), a derivative of pseudoginsenoside F11 from American ginseng, was observed to significantly potentiate the cytotoxic activity of Dox (Figure 1(a)). In this study, we explored the effects of ocotillol on the potency of Dox and its associated mechanism of action.

## 2. Materials and Methods

### 2.1. Materials

Ocotillol, prepared from American ginseng by Shandong Engineering Research Center for Natural Drugs, was obtained as white powder and had the molecular formula C_30_H_52_O_5_, MW 492. Purity of the compound used in present study was checked by HPLC and found to be higher than 98.5%. *In vitro*, Ocotillol, Dox (Zhejiang Hisun Pharmaceutical Co., Ltd., China), and pifithrin-*α* (Biyuntian, China) were dissolved in DMSO and stored at −20°C for less than one month before use. The vehicle, DMSO, was used as a control in all experiments at a maximum concentration of 0.1%. *In vivo*, Ocotillol and DOX were dissolved in 1% carboxymethyl cellulose sodium (CMCS) and 0.9% sodium chloride as proposed dose, respectively.

### 2.2. Cell Lines and Cell Culture

The human cancer cell lines A549, H1299, MCF7, and PC3 cells, were purchased from Cell Culture Center of Institute of Basic Medical Sciences, Chinese Academy of Medical Sciences. All the cancer cells were cultured in RPMI-1640 or DMEM medium supplemented with 10% fetal calf serum, penicillin (100 U/mL), and streptomycin (100 *μ*g/mL; Gibco BRL, NY, USA) and incubated at 37°C in a humidified air atmosphere containing 5% CO_2_. All cells were harvested in the exponentially growing phase.

### 2.3. Animals

Male nude mice (4~6 weeks old, BALB/c) were purchased from the Institute of Laboratory Animal Science, Chinese Academy of Medical Sciences, and Peking Union Medical College. All of the experiments were performed in accordance with the Guidelines for Care and Use of Experimental Animals of Experimental Animal Research Committee of Yantai University.

### 2.4. Cell Proliferation Assays

The viability of cell was evaluated using MTT assay as reported previously [20]. Briefly, cells were seeded into 96-well plates and then treated with tested articles at desired concentration for indicated time. MTT solution was added into the wells and incubated for 2 h. After the medium was removed, DMSO was added into each well. The plates were gently agitated until the color reaction was uniform and the OD_570_ was determined using a microplate reader (Wellscan MK3, Finland). Media-only treated cells served to indicate 100% cell viability, and the relative survival was defined as absorbance of treated wells divided by that of controls, in which the 50% inhibitory concentration (IC_50_) was defined as the concentration that reduced the absorbance by 50% of the controls.

### 2.5. Flow Cytometry Assay

Cell apoptosis was determined by flow cytometry (FCM) as previously reported [21]. A549 cells were seeded into 6-well plates, and treatments were initiated when cells were 60–70% confluent. Tested articles, diluted in medium, were added into the wells and incubated for 24 h, and then cells were harvested by digesting with trypsin/EDTA (Gibco BRL, NY, USA). After fixed with cold 70% ethanol, the cells were stained with propidium iodide (PI) solution (20 mg/mL PI and 20 mg/mL RNase A in PBS) for 30 min in 37°C. Samples were read on a Coulter Elite flow cytometer, and data were analyzed using Elite software program 4.0.

### 2.6. Hoechst 33342 Staining Assay

Effects of tested agents on apoptosis were visualized and quantified by immunofluorescence microscopy [22]. A549 cells were plated at 50,000 per well in 6-well cell culture plates with glass slides (Corning Incorporated, USA) and cultured overnight. Agents, diluted in medium, were added to desired concentrations. After 24 h exposure, cells were fixed with 3.7% formaldehyde in PBS for 10 min and stained with Hoechst 33342 solution (10 *μ*g/mL). The slides were washed twice in PBS and fixed onto the microscopic slide. The cell images were taken with a Kodak fluorescence microscope, and the number of apoptosis cells was counted in random fields for at least 1000 cells each group.

### 2.7. Quantitative Reverse Transcription Polymerase Chain Reaction

Cells were lysed in TRIzol (Invitrogen, USA), and total RNA was prepared as previously described [21]. After reverse transcription using a first-strand cDNA synthesis kit (Fermentas, USA), the cDNA was subjected to real-time polymerase chain reaction (RT-PCR) assays using the ABI 7500 RT-PCR System. Primer sequences were as follows: *PUMA*, F: 5′-CCT GGA GGG TCC TGT ACA ATC T-3′, R: 5′-GCA CCT AAT TGG GCT CCA TCT-3′; *BAX*, F: 5′-TGG AGC TGC AGA GGA TGA TTG-3′, R: 5′-AAA CAT GTC AGC TGC CAC TCG-3′; *PIG3*, F: 5′-TTG AGG CAT CTG GAC ATG TG-3′, R: 5′-GGG TCA ATC CCT CTG GGA TAG-3′; *GADPH (control)*, F: 5′-CAT GTT CCA ATA TGA TTC CAC C-3′, R: 5′-GAT GGG ATT TCC ATT GAT GAC-3′.

### 2.8. Western Blotting Assay

Cells were collected and prepared for the lysates after incubation, and then the cell lysates were performed to detect the target protein as previously described [21]. Briefly, total cellular proteins were electrophoresed on 10% SDS-polyacrylamide gels, and then the proteins were transferred to a PVDF membrane (Millipore, USA). After block, the membrane was incubated overnight with primary antibodies to P53 (Do-1, USA) and *β*-actin (C4, USA). The membranes were washed with TBS-0.05% Tween 20 for 5 min thrice and incubated with secondary antibodies and then visualized with an enhanced chemiluminescence detection kit (Amersham Life Sciences, USA). The film was developed, and the scanned pictures were shown.

### 2.9. Xenograft Tumor Model in Nude Mice

Male nude mice were introduced to establish xenograft tumor models of A549 as previously described [20]. Briefly, A549 cells were subcutaneously injected at the dorsum with 5 × 10^6^ cells in 0.1 mL, and treatment was started when the tumors reached an average volume of 100~300 mm^3^. Animals were randomized into 5 groups with 6 mice each group: (a) vehicle; (b) 10 mg/kg ocotillol; (c) 1.5 mg/kg Dox; (d) 10 mg/kg ocotillol plus 1.5 mg/kg Dox. Ocotillol was administrated by oral gavage, and Dox was injected intraperitoneally, which were at a volume of 10 mL/kg based on individual body weight. The mice were checked daily for toxicity/mortality relevant to treatment, and the tumor was measured with a caliper twice a week for up to endpoint days. The tumor volume in mm^3^ was calculated by the formula: volume = (width)^2^  ×  length/2, and the tumor growth curve was presented.

### 2.10. Data Analysis and Statistics

The results were presented as mean ± SD. Comparisons between more than 2 groups were performed by analysis of variance (one-way ANOVA), followed by Students *t*-test. The level of statistical significance was defined as *P* ≤ 0.05, unless indicated otherwise.

## 3. Results

### 3.1. Ocotillol Enhanced the Cytotoxic Activity of Dox in p53 Wild-Type Cancer Cells

**Figure 2:**
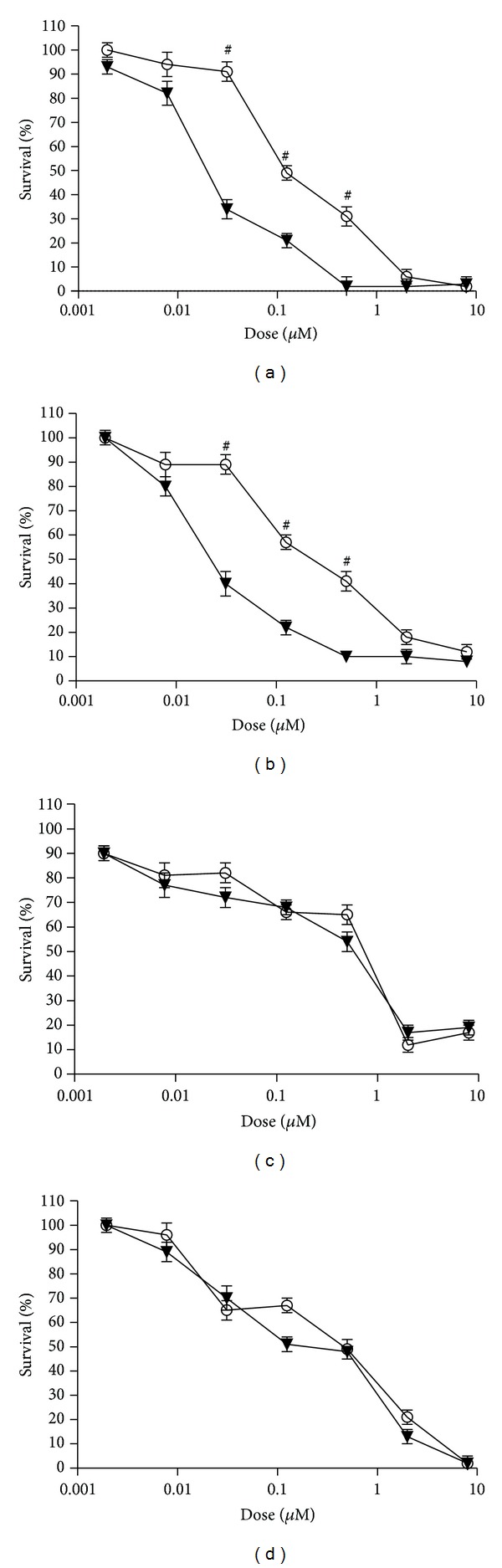
The effects of ocotillol on the cytotoxic activity of Dox in human cancer cells. A549 cells (a), MCF7 cells (b), PC3 cells (c), and H1299 cells (d) were seeded into 96-well plate and treated with Dox (○) or Dox + ocotillol (*▼*) as indicated. The cell viability was detected by MTT assay after 72 h incubation. ^#^ *P* < 0.05, compared with Dox group.

A series of MTT assays were performed to explore the effect of ocotillol on the anti-tumor activity of Dox in four cancer cells, such as A549, MCF7, PC3, and H1299. After 72 h incubation, Dox did display robust cytotoxic effect against all the tested cells *in vitro* (Figure 2 and Table 1). Interestingly, cotreatment with ocotillol at concentration of 5 *μ*M, which alone had no effect on cell proliferation, enhanced the cytotoxic effect of Dox in A549 and MCF7 cells (Figures 2(a) and 2(b)). The enhanced effects, however, were not observed in p53-null cells, such as PC3 and H1299 cell lines (Figures 2(c) and 2(d)). The IC_50_ values were calculated and shown in Table 1.

### 3.2. Ocotillol Potentiated Dox-Induced Cell Apoptosis

**Figure 3:**
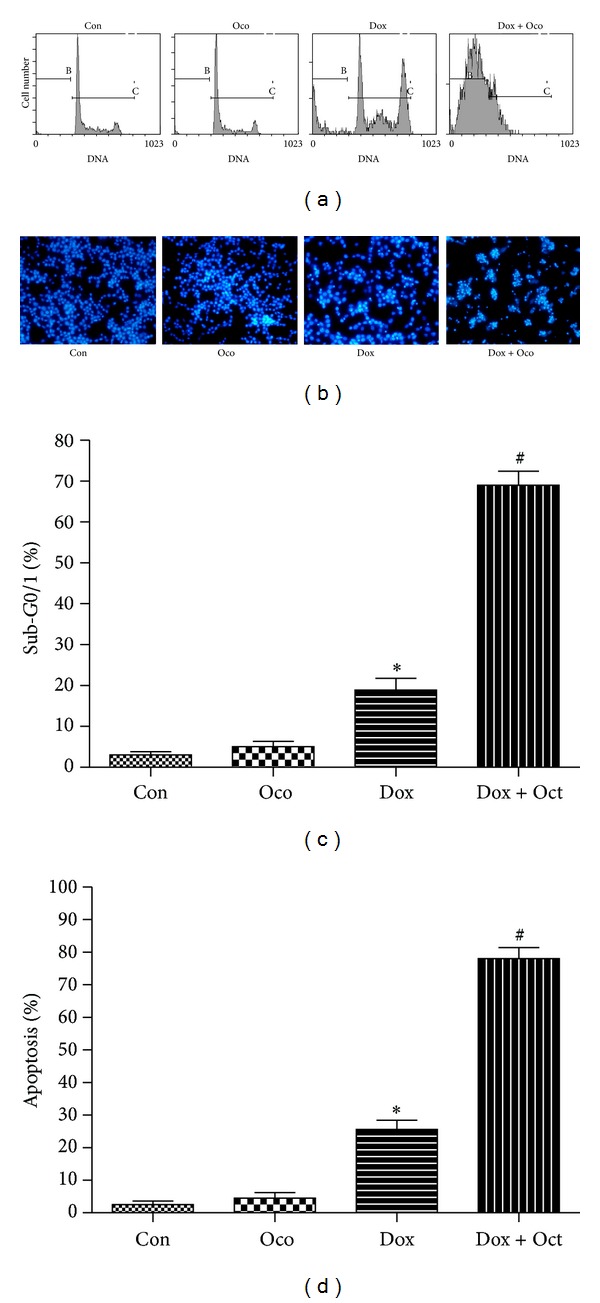
The effects of ocotillol on the cell apoptosis induced by Dox in A549 cells. A549 cells were seeded into 6-well plate and treated with Dox (0.5 *μ*M), Oco (0.5 *μ*M), or the combination for 24 h. The cells were then subjected to flow cytometry assay (a) to determine the percentages of apoptotic cells (c) or to Hoechst staining assays (b). At least 1000 cells were randomly chosen, and the numbers of apoptosis cells (determined by the morphologic observation) were counted (d). *P < 0.05, compared with control group; ^#^ *P* < 0.05, compared with Dox group.

FCM and Hoechst staining assays were then performed to detect the effect of Dox-O on cell apoptosis in A549 cells. As shown by FCM, the ratio of cells in sub-G0 phase, in which they were considered to be undergoing apoptosis, was significantly increased after Dox treatment for 24 h (Figures 3(a) and 3(c)). Ocotillol (5 *μ*M) alone had no effect on apoptosis but dramatically increased the ratio of cells in sub-G0 after being cotreated with Dox (*P* < 0.01, compared with the Dox only group). Similar results were found in Hoechst staining assay (Figure 3(b)), in which apoptotic cells were observed with characteristic morphologic characteristics, such as nucleic shrinkage. Apoptosis increased after Dox treatment for 24 h (*P* < 0.01, compared to control group); cells cotreated with Dox-O showed dramatically increased apoptosis (Figure 3(d), *P* < 0.01, compared to the Dox only group). Ocotillol alone had no effect on apoptosis at the tested concentration.

### 3.3. Dox with Ocotillol Enhanced the Activation of p53

**Figure 4:**
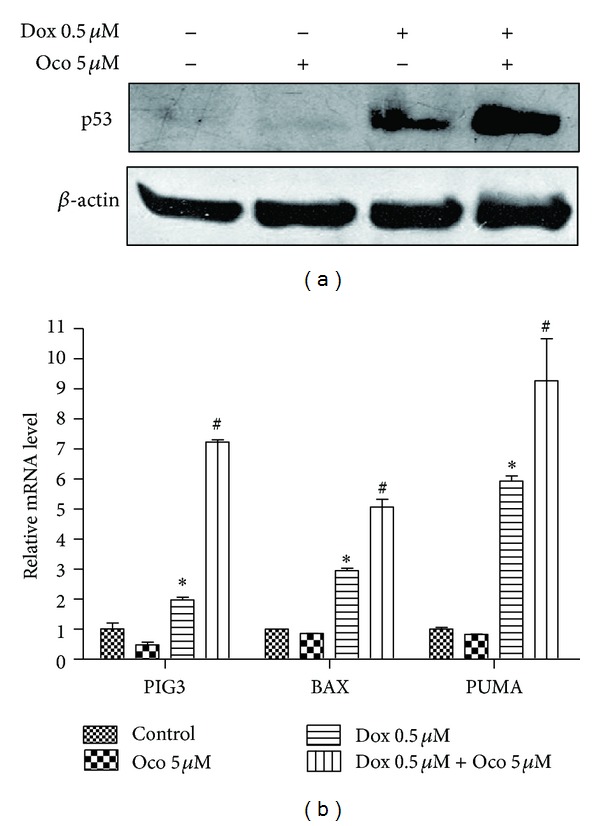
The effects of ocotillol on Dox-induced p53 activation. A549 cells were seeded into 6-well plate and treated with Dox (0.5 *μ*M), Oco (0.5 *μ*M), or the combination for 24 h. The cells were then lysed for immunoblotting to measure expression levels of p53 (a) or lysed for qRT-PCR assays to determine the *BAX*, *PUMA*, and *PIG3* mRNA levels. Data were depicted as average ± SD values of 3 determinations. *P < 0.05, compared with control group; ^#^ *P* < 0.05, compared with Dox group.

Dox dramatically increased p53 protein levels in A549 cells (Figure 4(a)), which in turn increased the induction of its downstream target genes, such as *BAX*, *PUMA*, and* PIG3* (Figure 4(b), *P* < 0.01, compared with control group). However, DOX-O activated p53 to a greater extent, which enhanced mRNA expression of *PUMA*, *BAX*, and *PIG3* (Figure 4(b), *P* < 0.01, compared with Dox alone group). Ocotillol at tested concentration had no obviously effect on the mRNA expression of *PUMA*, *BAX* but decreased mRNA expression of *PIG3*.

### 3.4. Ocotillol Enhanced Potency of Dox in a p53-Dependent Pathway

**Figure 5:**
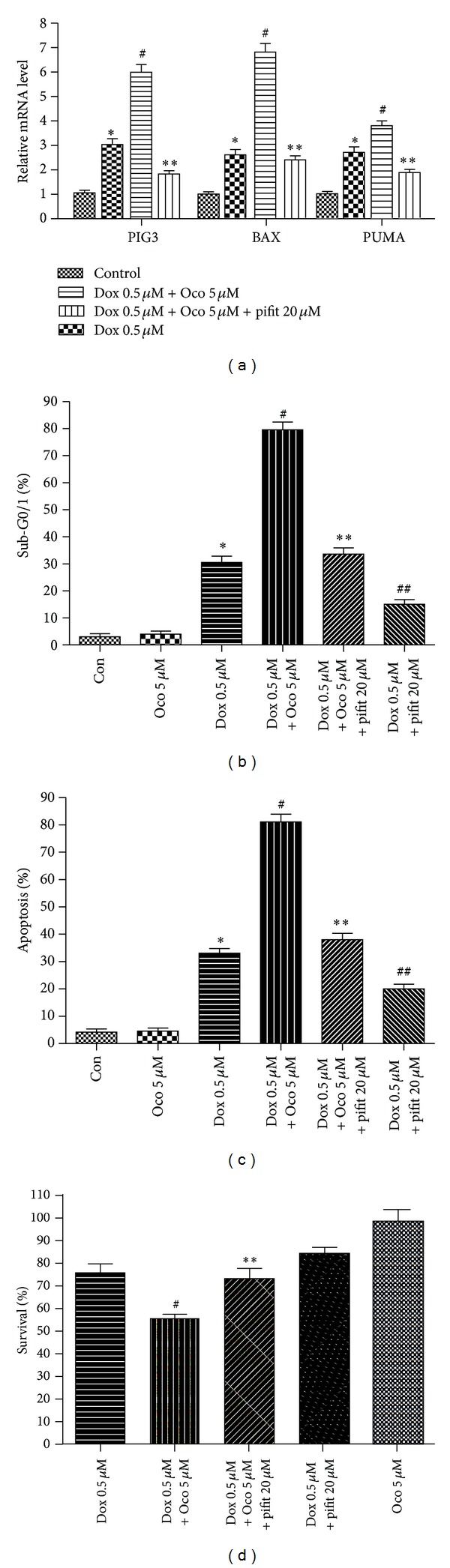
The effects of pifithrin-*α* on the cytotoxic activity of Dox-O in A549 cells. (a) A549 cells were seeded into 6-well plate and treated with as indicated for 24 h. The cells were then lysed for qRT-PCR assays to determine the *PUMA*,* PIG3*, and *BAX* mRNA levels. (b) and (c) A549 cells were treated as indicated for 24 h and subjected to flow cytometry (b) or Hoechst staining (c) to determine percentages of apoptotic cells. (d) A549 cells were treated as indicated, and the cell viability was detected by MTT assay after 24 h incubation. *P < 0.05, compared with control group; ^#^ *P* < 0.05, compared with Dox group. **P < 0.05, compared with Dox plus ocotillol group; ^##^ *P* < 0.05, compared with Dox group.

Pifithrin-*α*, a p53 inhibitor, is often used to explore the p53 signal pathway [23]. As shown in Figure 5, the increased proapoptosis genes induced by Dox-O, such as *BAX*, *PUMA*, and *PIG3*, were significantly attenuated by being coincubated with pifithrin-*α* (Figure 5(a), *P* < 0.01, compared with Dox-O group). FCM (Figure 5(b)) and Hoechst staining (Figure 5(c)) showed pifithrin-*α* to significantly suppress the increased apoptosis in the Dox-O group (*P* < 0.01, compared with Dox-O group). As a result, the enhanced cytotoxic activity of Dox-O was dramatically attenuated when cotreated with pifithrin-*α* (Figure 5(d), *P* < 0.01, compared with Dox-O group).

### 3.5. Ocotillol Potentiated the Anti-Tumor Activity of Dox in Xenograft Tumor Model

**Figure 6:**
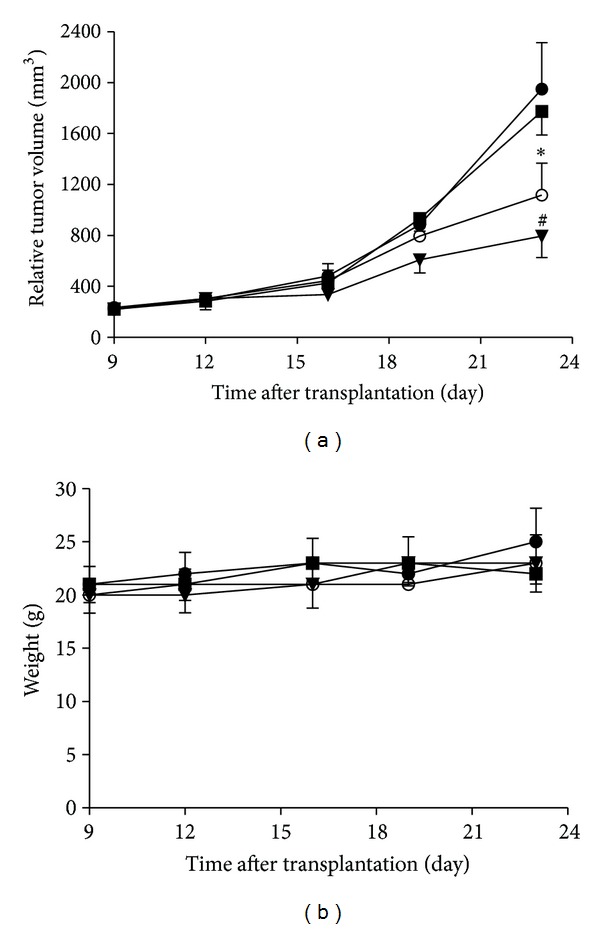
Effect of ocotillol on the antitumor activity of Dox on A549 xenograft tumors in nude mice. Tumor diameter was serially measured with a vernier caliper, the relative tumor volume was calculated, and the growth curve was drawn (a). The body weight changes of animals were recorded and shown (b). Vehicle (●); 10 mg/kg ocotillol (■); 1.5 mg/kg Dox (○); 1.5 mg/kg Dox plus 10 mg/kg Ocotillo (*▼*). *P < 0.05, compared with control group; ^#^ *P* < 0.05, compared with Dox group.

A549 xenograft tumor was established, and the mice were treated with vehicle, ocotillol, Dox or Dox-O. As shown in Figure 6(a), ocotillol at dose of 10 mg/kg, which alone had no obvious effect on the tumor growth, could significantly increase the anti-tumor activity of Dox administrated every three days at dose of 1.5 mg/kg (*P* < 0.01, compared with Dox alone group). Little weight loss was observed (Figure 6(b)), and no animal was dead in all treated groups.

## 4. Discussions

Cancer was one of the major causes of death in the world, and its first-line and most salient treatment strategy still was the cytotoxic agent-based chemotherapy [2, 24]. Although Dox-based chemotherapy could increase patients' survival, side effects and acquisition of drug resistance severely limited its clinical effectiveness [25]. Therefore, novel therapeutic strategies, in which cotreatment with a sensitizer could reduce the cumulative doses of Dox by enhancing the anti-tumor activities, needed to be developed. Here, we provided the first evidence that ocotillol could potentiate the cytotoxic activity of Dox via p53-dependent apoptosis. 

In current study, ocotillol was observed to induce higher sensitization to Dox in p53 wild-type cell lines, not in p53-null cells (Figure 2), which indicated the enhancement of ocotillol on potency of Dox might be in a p53-dependent manner. Based on our previous data, ocotillol did not aggravate the cardiotoxicity of Dox in H9C2 cells [17]. Indeed, ocotillol could significantly enhance the anti-tumor activity of Dox in xenograft tumor models without apparently increasing its toxic effect, which was primarily evaluated by the animal survival and body weight (Figure 6). Dox-O combination therapy, therefore, could exert more robust anti-tumor effects and does not increase toxic effects, which might in turn decrease the cumulative toxic effects through reducing Dox dosage. 

Inducing apoptosis was a common mechanism of anti-cancer drugs [26], in which the apoptotic cells were easily detected by several methods, such as FCM and Hoechst staining [27]. Stained apoptotic cells are showed with less nuclear PI dye and could be recorded as sub-G0 phase in FCM assay, and morphologic characteristics of apoptotic cells such as nucleus shrinkage and DNA breakage were shown after stained with Hoechst 33342. In the current study, both FCM and Hoechst staining showed proportions of apoptotic cells to be dramatically increased in the Dox-O group compared with the Dox alone group (Figure 3), indicating that ocotillol potentiated Dox cytotoxicity via promoting the cancer cells undergoing apoptosis.

As a transcription factor, p53 played an important role in many cancer drugs' anti-tumor effects, including both “cytotoxic drugs” and “target drugs” [28]. Doxorubicin, paclitaxel, etoposide, and cisplatin reportedly exerted cytotoxic activities through activating p53, resulting in increased expression of its target genes, such as *PUMA*, *PIG3*, and *BAX* [29]. In this study, mRNA expression *PUMA*, *PIG3* and *BAX* were increased following p53 activation after Dox treatment. Dox-O was seen to further increase mRNA expression of *PUMA*, *PIG3* and *BAX* (Figure 4), which were all known to be proapoptosis genes, induction of which led cells to undergo apoptosis. As a result, MTT assay detected this as decreased cell viability. 

To verify that ocotillol's effect on the cytotoxicity of Dox was through p53 pathway, the p53 inhibitor pifithrin-*α* was used to block p53 transcription activity [23]. Indeed, pifithrin-*α* could significantly attenuated the increase of pro-apoptosis genes induced by Dox-O (Figure 5). As a result, the increased Dox-induced apoptosis in both the presence and absence of ocotillol was dramatically attenuated by co-administration with pifithrin-*α*. Consequently, the MTT assay showed that pifithrin-*α* repressed Dox cytotoxicity with and without ocotillol. The finding was consistent with the fact that Dox displayed anti-cancer activity through, or at least partially through, activating p53 [29]. All of these results showed clearly p53 played the major role in the effect of ocotillol on Dox potency, The exact molecular mechanisms of action, how Dox with ocotillol could further activate p53, and which regulator protein was involved were still needed to be explored.

## 5. Conclusion

We here reported for the first time that ocotillol enhanced Dox cytotoxicity apparently by inducing p53-dependent apoptosis. This implied that use of ocotillol with Dox could be an improved therapeutic strategy.

## Figures and Tables

**Table 1:** Inhibitory effects of Dox, Oco, or the combination on tumor cell lines.

Cell line	p53 status		IC_50_ (*μ*M)	
		Dox	Dox + Oco 5 *μ*M	Oco
A549	Wild type	0.16 ± 0.01	0.04 ± 0.01	>50
MCF7	Wild type	0.24 ± 0.03	0.05 ± 0.01	>50
PC3	Null	0.27 ± 0.03	0.24 ± 0.02	>50
H1299	Null	0.22 ± 0.04	0.16 ± 0.01	>50

## References

[1] Jemal A, Bray F, Center MM, Ferlay J, Ward E, Forman D (2011). Global cancer statistics. *CA Cancer Journal for Clinicians*.

[2] Lal R, Enting D, Kristeleit H (2011). Systemic treatment of non-small-cell lung cancer. *European Journal of Cancer*.

[3] Beil DR, Wein LM (2002). Sequencing surgery, radiotherapy and chemotherapy: insights from a mathematical analysis. *Breast Cancer Research and Treatment*.

[4] Carvalho C, Santos RX, Cardoso S (2009). Doxorubicin: the good, the bad and the ugly effect. *Current Medicinal Chemistry*.

[5] Drummond DC, Meyer O, Hong K, Kirpotin DB, Papahadjopoulos D (1999). Optimizing liposomes for delivery of chemotherapeutic agents to solid tumors. *Pharmacological Reviews*.

[6] Scott JM, Khakoo A, MacKey JR, Haykowsky MJ, Douglas PS, Jones LW (2011). Modulation of anthracycline-induced cardiotoxicity by aerobic exercise in breast cancer: current evidence and underlying mechanisms. *Circulation*.

[7] Minotti G, Menna P, Salvatorelli E, Cairo G, Gianni L (2004). Anthracyclines: molecular advances and pharmacologie developments in antitumor activity and cardiotoxicity. *Pharmacological Reviews*.

[8] Marine J-C (2010). Pharmacological rescue of p53 in cancer therapy: widening the sensitive tumor spectrum by targeting MDMX. *Cancer Cell*.

[9] Harris SL, Levine AJ (2005). The p53 pathway: positive and negative feedback loops. *Oncogene*.

[10] Qian H, Yang Y, Wang X (2011). Curcumin enhanced adriamycin-induced human liver-derived Hepatoma G2 cell death through activation of mitochondria-mediated apoptosis and autophagy. *European Journal of Pharmaceutical Sciences*.

[11] Spina A, Sorvillo L, Di Maiolo F (2012). Inorganic phosphate enhances sensitivity of human osteosarcoma U2OS cells to doxorubicin via a p53-dependent pathway. *Journal of Cellular Physiology*.

[12] Yuan X-W, Zhu X-F, Huang X-F (2007). Interferon-*α* enhances sensitivity of human osteosarcoma U2OS cells to doxorubicin by p53-dependent apoptosis. *Acta Pharmacologica Sinica*.

[13] Schwartsmann G, Ratain MJ, Cragg GM (2002). Anticancer drug discovery and development throughout the world. *Journal of Clinical Oncology*.

[14] Karmazyn M, Moey M, Gan XT (2011). Therapeutic potential of ginseng in the management of cardiovascular disorders. *Drugs*.

[15] Qi L-W, Wang C-Z, Yuan C-S (2011). Ginsenosides from American ginseng: chemical and pharmacological diversity. *Phytochemistry*.

[16] Wang Z, Zheng Q, Liu K, Li G, Zheng R (2006). Ginsenoside Rh2 enhances antitumour activity and decreases genotoxic effect of cyclophosphamide. *Basic and Clinical Pharmacology and Toxicology*.

[17] Wang H, Yu P, Gou H (2012). Cardioprotective effects of 20(S)-ginsenoside Rh2 against doxorubicin-induced cardiotoxicity *in vitro* and *in vivo*. *Evidence-Based Complementary and Alternative Medicine*.

[18] Xie X, Eberding A, Madera C (2006). Rh2 synergistically enhances paclitaxel or mitoxantrone in prostate cancer models. *Journal of Urology*.

[19] Kim S-W, Kwon H-Y, Chi D-W (2003). Reversal of P-glycoprotein-mediated multidrug resistance by ginsenoside Rg3. *Biochemical Pharmacology*.

[20] Wang H, Li H, Zuo M (2008). Lx2-32c, a novel taxane and its antitumor activities *in vitro* and *in vivo*. *Cancer Letters*.

[21] Wang H, Ma X, Ren S, Buolamwini JK, Yan C (2011). A small-molecule inhibitor of MDMX activates p53 and induces apoptosis. *Molecular Cancer Therapeutics*.

[22] Lizard G, Deckert V, Dubrez L, Moisant M, Gambert P, Lagrost L (1996). Induction of apoptosis in endothelial cells treated with cholesterol oxides. *American Journal of Pathology*.

[23] Komarov PG, Komarova EA, Kondratov RV (1999). A chemical inhibitor of p53 that protects mice from the side effects of cancer therapy. *Science*.

[24] Rutkoski TJ, Raines RT (2008). Evasion of ribonuclease inhibitor as a determinant of ribonuclease cytotoxicity. *Current Pharmaceutical Biotechnology*.

[25] Appel JM, Nielsen D, Zerahn B, Jensen BV, Skagen K (2007). Anthracycline-induced chronic cardiotoxicity and heart failure. *Acta Oncologica*.

[26] Ahmad A, Sakr WA, Rahman KMW (2010). Anticancer properties of indole compounds: mechanism of apoptosis induction and role in chemotherapy. *Current Drug Targets*.

[27] Darzynkiewicz Z, Bruno S, Del Bino G (1992). Features of apoptotic cells measured by flow cytometry. *Cytometry*.

[28] Levine AJ, Oren M (2009). The first 30 years of p53: growing ever more complex. *Nature Reviews Cancer*.

[29] Brown CJ, Lain S, Verma CS, Fersht AR, Lane DP (2009). Awakening guardian angels: drugging the P53 pathway. *Nature Reviews Cancer*.

